# A Case Report on Hepatic Portal Venous Gas (HPVG)

**DOI:** 10.7759/cureus.30689

**Published:** 2022-10-26

**Authors:** Joseph Latham, Gayan Nanayakkara

**Affiliations:** 1 General Surgery, Withybush General Hospital, Haverfordwest, GBR

**Keywords:** portal venous gas, hepatic portal vein gas, hepatic portal venous gas, gi radiology, git endoscopy, pneumatosis intestinalis, gastric pneumatosis, haematemesis, hpvg

## Abstract

Hepatic Portal Venous Gas (HPVG) is the abnormal presence of gas in the portal venous system. It is associated with life-threatening conditions and is a sinister radiological sign. This case report aims to evaluate the significance of HPVG as a radiological sign. Our case involves a 49-year-old man who was admitted to the hospital following a one-day history of severe epigastric pain and haematemesis. Investigations showed extensive HPVG, gastric pneumatosis, a large retroperitoneal haematoma, and an obstructive lesion between the first and second part of the duodenum. Our patient was managed conservatively in the High Dependency Unit (HDU). A repeat Computerised Tomography (CT) scan showed successful resolution of the HPVG and gastric pneumatosis without any invasive intervention.

## Introduction

Hepatic Portal Venous Gas (HPVG) is the accumulation of gas in the portal vein and its branches. It is considered to be an ominous radiological sign, associated with life-threatening conditions [1-3]. This case report aims to evaluate the significance of HPVG as a radiological sign. Since the introduction of CT scans, there has been an increased frequency of benign cases of HPVG reported. This is largely thought to be due to the high sensitivity of CT scans, resulting in earlier and more frequent detection than previously [4]. The exact pathogenesis of HPVG is not well understood however, there are several theories: (1) An increased intraluminal pressure causes gas to escape from the bowel into the portal venous system via the mesenteric veins [1], (2) Intraluminal bacteria produce hydrogen gas which then diffuses into the portal venous system via the mesenteric veins [1], (3) Mucosal damage due to specific disease mechanisms causes gas to pass into the portal venous system via the mesenteric veins [1,2].

Several large studies have shown the broad spectrum of aetiological conditions associated with HPVG including, bowel ischaemia, Gastrointestinal Tract (GIT) obstruction, GIT infection, GIT perforation, and necrotizing pancreatitis [1-4].Management of HPVG is directed towards treating the underlying pathological cause and therefore the prognosis is dependent on the underlying cause [1,2].

## Case presentation

Our patient was a 49-year-old male with a background of Chronic Pancreatitis with a pseudocyst in the head of the pancreas, three renal transplants for renal failure secondary to resistant hypertension, Hereditary Hemorrhagic Telangiectasia (HHT), and Barrett’s Oesophagus. He presented to the Emergency Department with a one-day history of severe epigastric pain radiating to the back with associated nausea and haematemesis.

On examination, his heart rate was 155 beats per minute, his blood pressure was stable and he was afebrile. His abdomen was soft, but he had significant right upper quadrant and epigastric tenderness. Full blood count showed a haemoglobin drop by 15 g/dl from baseline. There was an Acute Kidney Injury (AKI) on the background of Chronic Kidney Disease (CKD). Liver Function Tests were unremarkable. Amylase and C-Reactive Protein (CRP) were mildly elevated.

**Figure 1:**
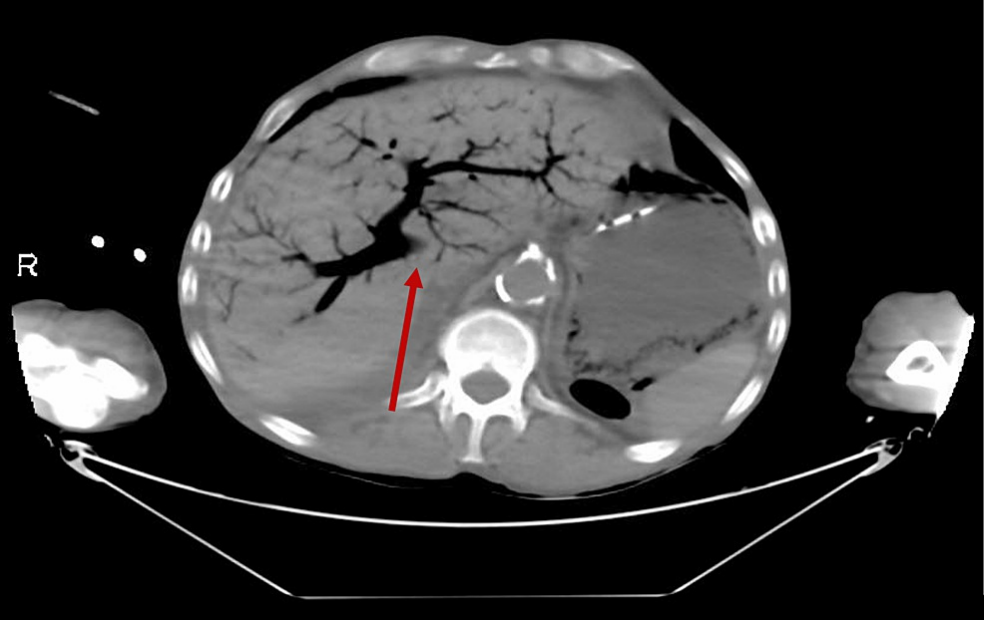
CT Abdomen/Pelvis (axial view) The image is showing HPVG (Hepatic Portal Venous Gas)

**Figure 2:**
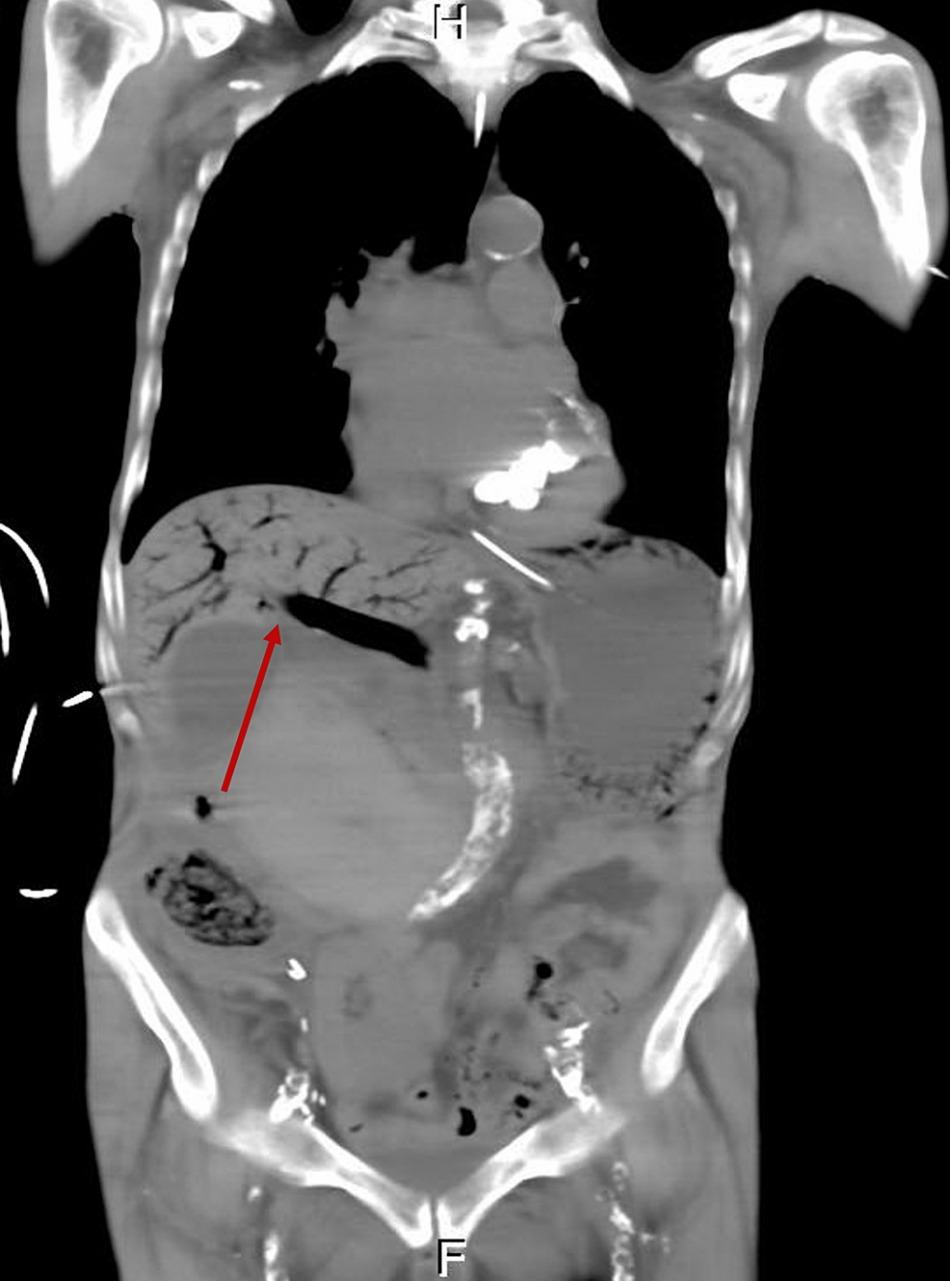
CT Abdomen/Pelvis (coronal view) The image is showing HPVG (Hepatic Portal Venous Gas)

Due to the renal function, a non-contrast CT abdomen and pelvis was performed. This showed ominous extensive aeroportia, air within the splenic and superior mesenteric vein, and gastric pneumatosis with a significantly dilated stomach (Figures 1, 2).

**Figure 3:**
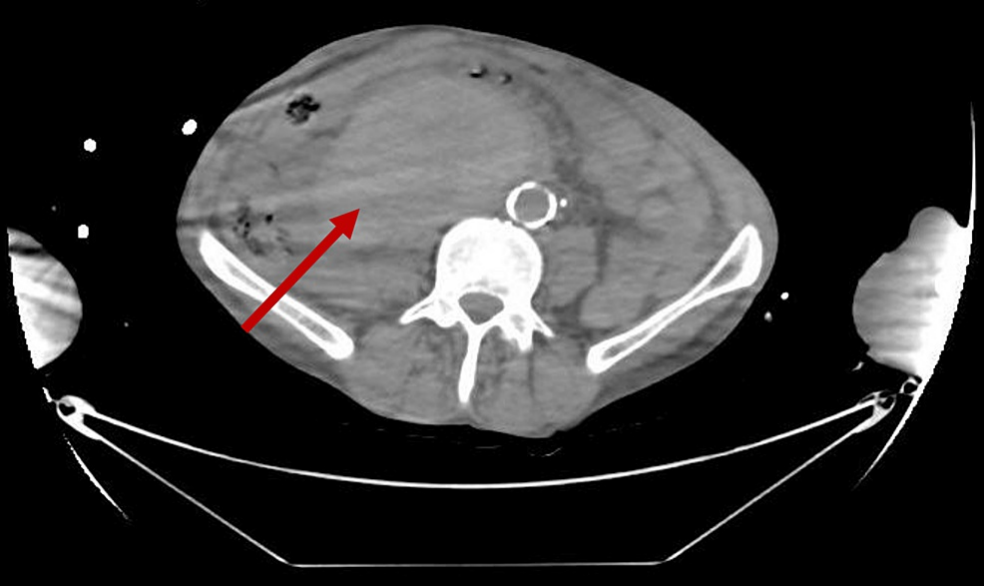
CT Abdomen/Pelvis (axial view) The red arrow is showing the haematoma

**Figure 4:**
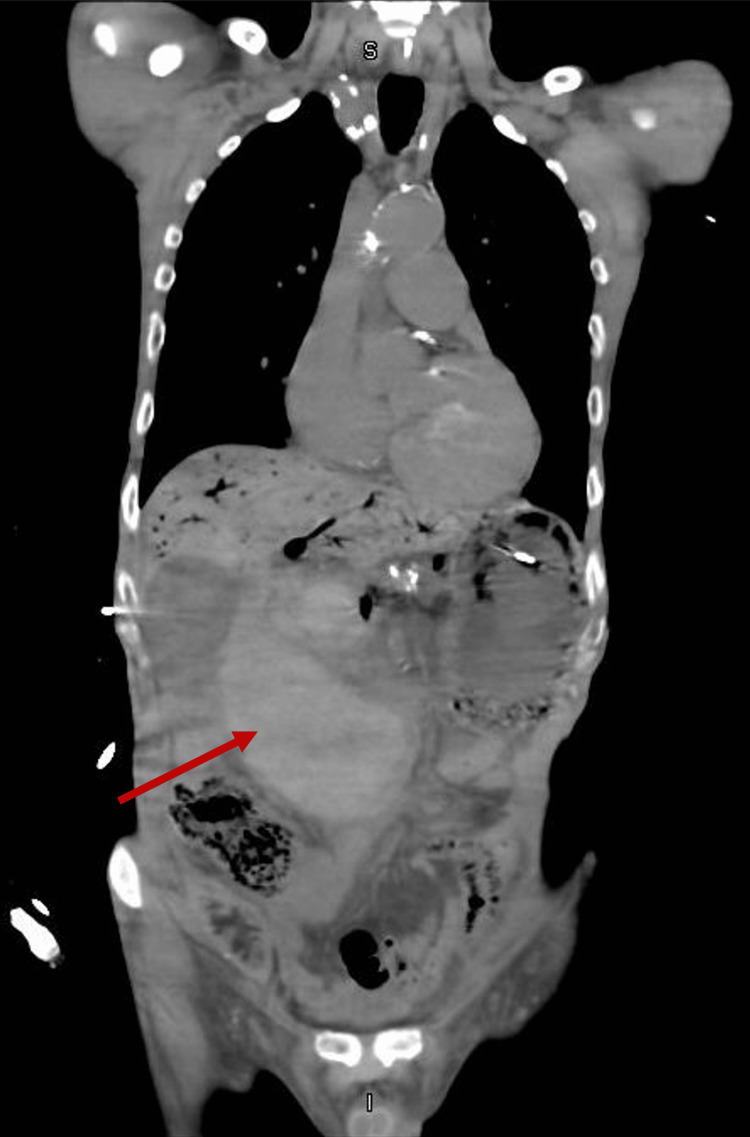
CT Abdomen/Pelvis (coronal view) The red arrow is showing the haematoma

Additionally, it showed, a large retroperitoneal mass, either a haemorrhagic tumour or massive submucosal haematoma extending down from the retroduodenal area, measuring at least 15 cm cephalo-caudally (Figures 3, 4]. 

**Figure 5:**
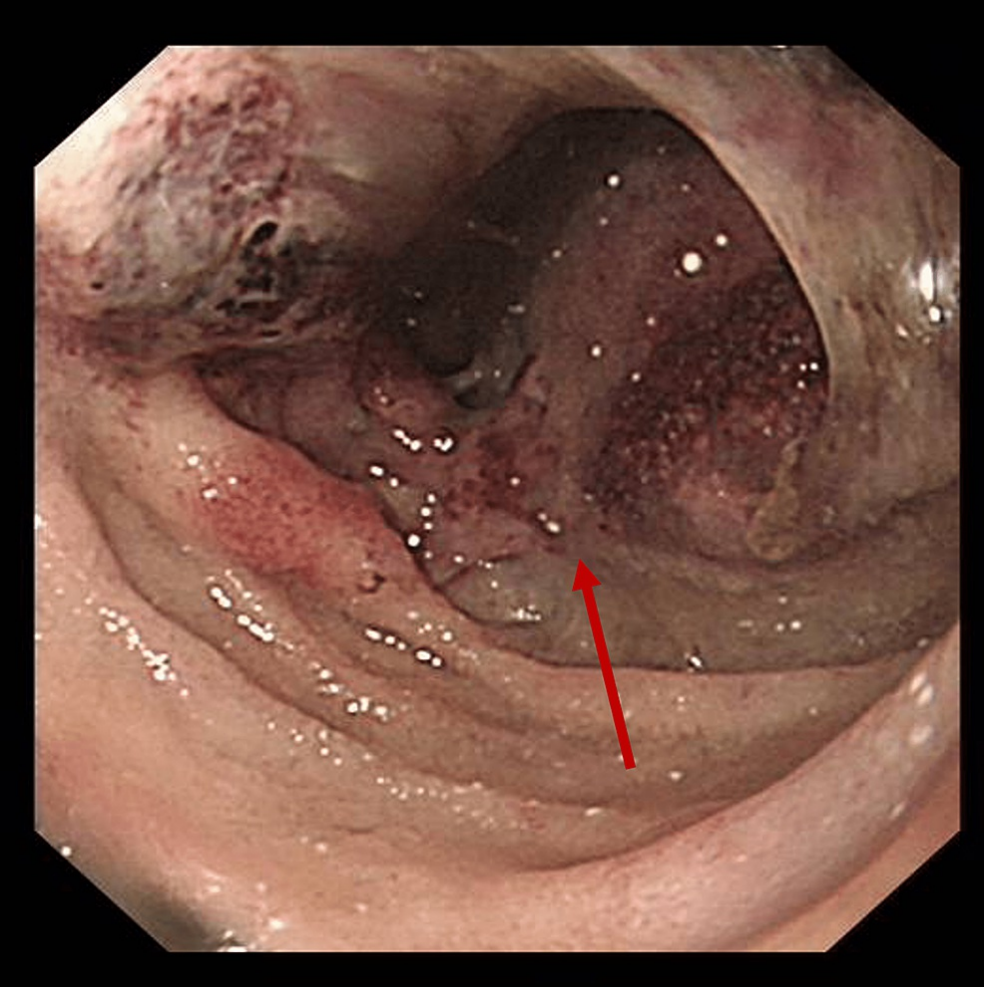
Endoscopy image of the obstructive lesion at the D1/D2 junction D1: the superior part of the duodenum; D2: the descending duodenum

Due to the haematemesis, an Oesophagogastroduodenoscopy (OGD) was performed. This showed a lot of coffee ground fluid in the stomach and oesophagus. The oesophagus was normal with no varices. The superior part of the duodenum (D1) was normal but there was an obstructive and inflamed lesion just after its junction with the descending duodenum (D2). The lesion was thought to be either malignant or a significant perforated duodenal ulcer with clots. However, there was no active spurting or oozing of blood to target any endotherapy (Figure 5). Histology later revealed mild, non-specific, chronic, inflammation of the duodenal mucosa but no evidence of malignancy. 

Initially, prior to the CT scan and gastroscopy, our patient was treated as an Acute Exacerbation of Chronic Pancreatitis due to the clinical presentation, blood results, and background. The patient received intravenous (IV) fluids, analgesia, and antiemetics whilst awaiting further investigations.

Given the patient's extensive co-morbidities, CT results, and gastroscopy findings, the patient was admitted to the high-dependency unit (HDU) for supportive management. The patient and family were consulted on the severity of the diagnosis and explained the poor overall prognosis. They were transfused several units of Red Blood Cells (RBC), started on IV antibiotics, and given an IV Proton Pump Inhibitor (PPI). 

**Figure 6:**
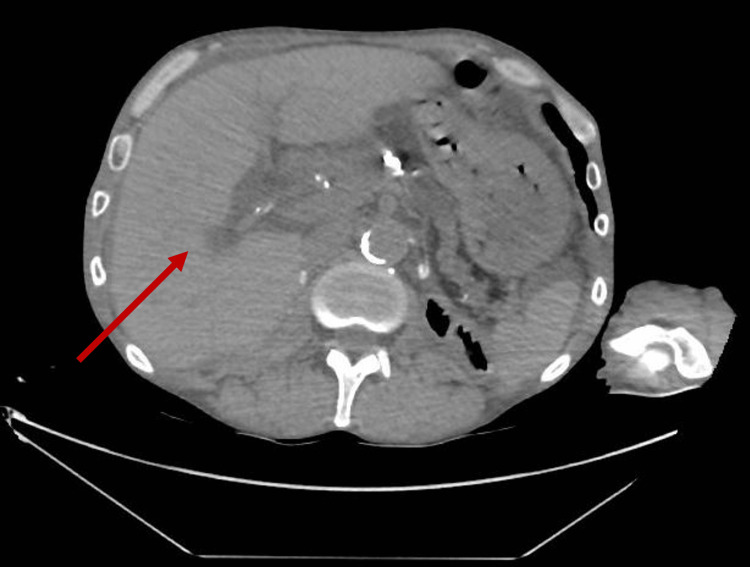
CT Abdomen/Pelvis (axial view) The image is showing resolution of the previous HPVG (Hepatic Portal Venous Gas)

**Figure 7:**
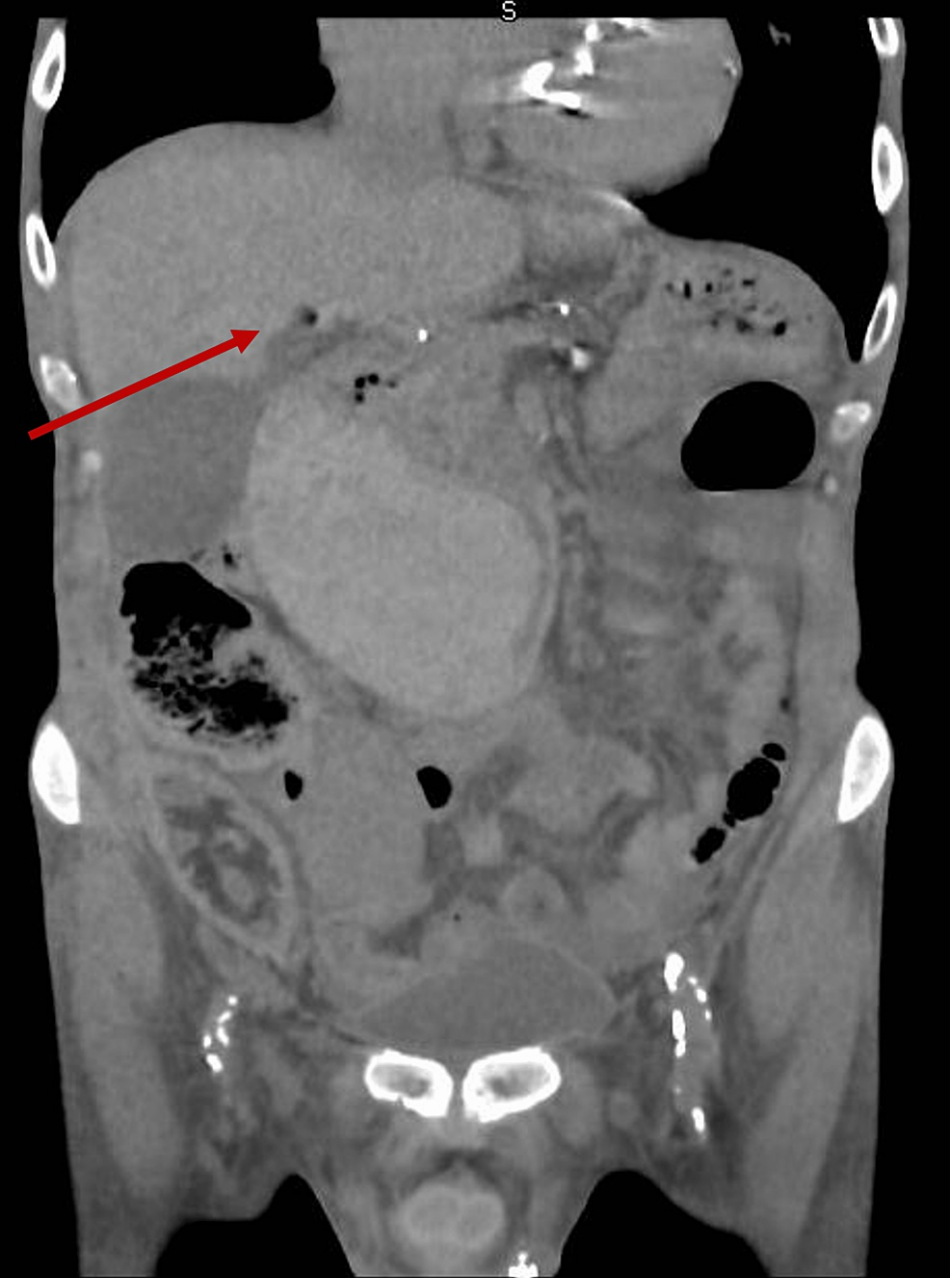
CT Abdomen/Pelvis (coronal view) The image is showing resolution of the previous HPVG (Hepatic Portal Venous Gas)

A repeat CT four days later showed a significant decrease in the volume of portal venous gas and full resolution of the previously demonstrated gastric pneumatosis (Figures 6, 7]. However, due to the patient’s declining kidney function and complex background of multiple renal transplants, the patient was transferred to a tertiary renal transplant centre.

Our patient was followed up with a repeat gastroscopy four months later which showed Barrett’s oesophagus only. There was no evidence of any duodenal lesion. A repeat CT was also performed at the same time which showed stable appearances of the retroperitoneal haematoma and there was no evidence of HPVG or gastric pneumatosis. Sadly, our patient passed away from an unrelated cause later in the year.

## Discussion

Following OGD, histology, and CT scan there was no definitive evidence to explain the HPVG. The general impression was that the patient had bled from the duodenum into the pancreatic head pseudocyst and the HPVG was secondary to this. One explanation was that the patient had bled, possibly from a duodenal telangiectasia, into the pancreatic pseudocyst in the head creating the large haematoma. This haematoma may have caused pressure necrosis on the duodenum leading to the stricture seen on an endoscopy, which subsequently caused HPVG. Another explanation is that a duodenal ulcer eroded the gastroduodenal artery causing bleeding into the pancreatic pseudocyst resulting in the large haematoma and the HPVG was caused because of mucosal damage from the ulcer. Regardless, our patient made a full recovery without any invasive intervention. This is contrary to previous literature surrounding HPVG and more in line with emerging literature. 

HPVG is still considered to be a rare and potentially life-threatening radiological sign. It was first documented by Wolfe and Evans [5] in patients with necrotising enterocolitis and has since been reported in a broad spectrum of life-threatening and benign conditions [1,5]. Its overall prognosis is not well documented. A study by Leibman et al. in 1978 [2] showed the overall mortality associated with HPVG to be 75% whilst, a more recent study by Koizumi et al. in 2018, demonstrated an overall in-hospital mortality of 27.3% [3]. It is thought that this discrepancy in mortality rates is likely due to detection bias as a result of the increased frequency of CT scans [1,4,6].

Leibman et al. also reviewed 64 cases of HPVG finding that it was associated with the following conditions: bowel necrosis (72%), ulcerative colitis (8%), intra-abdominal abscess (6%), small bowel obstruction (3%). and gastric ulcer (3%) [2]. Similarly, Koizumi et al. found that HPVG was associated with bowel ischaemia in 53% of the reviewed cases. The study went on to demonstrate the prognosis of each associated condition. They concluded that HPVG with bowel ischaemia had an in-hospital mortality of 26.8%, with GIT obstruction or dilatation at 31.1%, GIT perforation at 33.3%, GIT infection at 13.6%, and GIT sepsis at 56.4% [3].

A literature search provides similar cases to ours. One study by Dong et al. describes the successful conservative management of a patient with HPVG secondary to acute pancreatitis. The CT images showed HPVG with an inflamed oedematous pancreas and raised pancreatic enzymes. A follow-up CT showed resolution following conservative management [7]. Our patient had a background of chronic pancreatitis and he presented with epigastric pain and mildly raised amylase. Although this was not our leading differential it does share similarities.

Likewise, a study by Ginesu et al. reports an 82-year-old man developing HPVG post left-hemicolectomy for stenosing tumour in the descending colon. HPVG resolved completely following conservative management. This report illustrates the importance of recognising HPVG as a radiological sign separate from the underlying cause and therefore highlights the varying prognostics depending on the associated condition [8].

Another case by Nevins et al. describes the management of a 93-year-old gentleman with HPVG secondary to bowel ischaemia who was successfully managed conservatively without surgical intervention. However, this is an extremely rare example and requires further investigation [9].

Yoo et al. explored the outcomes of surgical management versus conservative management in patients with HPVG. They separated patients into two groups, those requiring surgical intervention and those requiring conservative management. Of the surgical group, 50% were not suitable for theatre due to haemodynamic instability or personal refusal. For these patients, there was 100% mortality. Of those who went to the theatre, there was a 40% mortality within 24 hours. This is compared to the group who were considered suitable for conservative management, of which only 12.5% died. This study stresses the importance of appropriately identifying which patients are suitable for theatre and which are for conservative management [4].

## Conclusions

In conclusion, HPVG is a radiological sign associated with a broad spectrum of conditions. Management of HPVG involves treating the underlying cause and therefore the prognosis of HPVG is dependent on the underlying condition itself. Additionally, due to the increased use of CT scanning in medicine, there have been more cases of benign HPVG and thus making the radiological sign less ominous than previously considered.
